# Human Herpesvirus 8 in Healthy Blood Donors, Argentina

**DOI:** 10.3201/eid1601.090893

**Published:** 2010-01

**Authors:** Celeste L. Pérez, Mónica I. Tous, Norma Zala, Sofía Camino

**Affiliations:** Administración Nacional de Laboratorios e Institutos de Salud, Buenos Aires, Argentina (C.L. Perez, M.I. Tous); Hospital de Enfermedades Infecciosas “Francisco J Muñiz,” Buenos Aires (N. Zala, S. Camino)

**Keywords:** Herpesvirus 8, human, DNA, blood, KSHV, saliva, viruses, letter

**To the Editor:** Human herpesvirus 8 (HHV-8), or Kaposi sarcoma–associated herpesvirus, is associated with malignant disorders such as Kaposi sarcoma, primary effusion lymphoma, and multicentric Castleman disease. Although HHV-8 does not necessarily cause life-threatening infection in healthy persons, it causes more severe infection in those who are immunocompromised, such as organ recipients and HIV-infected persons.

HHV-8 has been found in a number of clinical specimens (blood, saliva, and semen) from persons with HHV-8 related diseases (*1*,*2*). Identification of infectious virus in lymphocytes from a healthy blood donor and evidence that HHV-8 might be transmitted by blood has raised concern about the safety of the blood supply (*3*,*4*). Few studies have detected viral DNA in blood samples of blood donors from areas with low HHV-8 prevalence (*5**–**7*)*.* During January 2000 and December 2002, the Virology Department, National Institute of Infectious Diseases, Administración Nacional de Laboratorios e Institutos de Salud, “Dr C G. Malbrán” conducted an HHV-8 serosurvey of 6 blood banks from 5 South American regions and found overall seroprevalence to be 3.7% (range 1.9%–6.7%). The 6.7% seroprevalence from a blood bank in Buenos Aires city was substantially higher than that of other blood banks (*8*)*.*

From July 2004 through January 2005, to look for the virus in blood and saliva, we conducted the study reported here, an HHV-8 survey at the same blood bank. A total of 577 volunteer blood donors (431 men and 146 women), mean age 39 years (range 17–76 years), were enrolled at the Hemotherapy Service, Hospital of Infectious Diseases “Francisco Javier Muñiz.” The protocol was approved by the Teaching and Research Committee.

Serum and whole blood were collected from all 577 donors, and paired blood–saliva samples were obtained from 394. Serum samples were routinely tested for hepatitis B and C viruses, HIV, human T-lymphotropic viruses I and II, *Treponema pallidum, Brucella* spp., and *Trypanosoma cruzi*; results were used to determine associations between HHV-8 and these agents. Specimens were stored at –20°C until serologic and molecular investigation at the Virology Department, National Institute of Infectious Diseases.

Serologic screening for HHV-8 infection was performed by indirect immunofluorescence assay by using lytically induced cells; serum samples were diluted 1:40 (*8*). Then 45 blood and 39 paired blood–saliva samples from HHV-8-seroreactive donors were investigated for viral genome by open reading frame 26 nested PCR. DNA was purified from 0.3 mL of whole blood by using FlexiGene DNA Kit (QIAGEN, Gmbh, Hilden, Germany); concentrations and quality were measured with a UV spectrophotometer, and 1 μg was used for PCR*.* The QIAamp DNA Mini Kit (QIAGEN, Gmbh,) was used to obtain DNA from 0.2-mL saliva samples. Crude pellets were resuspended in 20µL of Tris EDTA, pH 8, then 5 µL were added to the PCR. Quality of DNA isolated from negative PCR samples was tested by amplifying the human housekeeping gene β-globin. In addition, inhibitors were investigated by adding the minimum viral DNA amount detected by our nested PCR, previously assessed by 10-fold serial dilutions of DNA from body cavity–based lymphoma 1 cells. The results are expressed as percentages, 95% confidence intervals (CIs), and proportions (positive/total). When necessary, the associations between variables were tested by means of χ^2^ or logistic regression. Significance was defined as p<0.05. Data were analyzed by using the Epidat 3.0 program, available from www.paho.org.

Positive immunofluorescence assay results were obtained for 45 (7.79%) of the 577 blood donors; seroprevalence was independent of gender (p = 0.8) and increased with age (odds ratio 1.04, 95% CI 1.01–1.07, p = 0.028). No association was found between HHV-8 and seroreactivity to the infectious agents tested (p = 0.3438). HHV-8 DNA was found in 3 seroreactive blood donors: 1 in saliva only and 2 in blood and saliva. Of the 45 HHV-8 seropositive samples, 38 were nonreactive to any infectious agents tested in the blood bank. One donor was seroreactive for hepatitis B.

In summary, we found HHV-8 in blood and saliva of blood donors even in an area where the virus is not endemic. Seroprevalence for HHV-8 was similar to that previously reported (*8*). Also, low viral loads might be undetectable by PCR but high enough to cause an infection with usual volumes of blood used in transfusions (*9*)*,* especially when the hemoderivatives are given to immunocompromised recipients. This study was done in a blood bank from a hospital for infectious diseases in which the recipient population consisted of numerous HIV patients (*10*). It is a concern that these patients could have received blood infected with HHV-8. The fact that saliva samples were also positive is consistent with previously reported findings (*1*,*2*) and might indicate that the virus is active at a site from which samples are easier to obtain and in which the virus easier to detect than the bloodstream. This study provides further evidence that blood transfusion carries a potential risk for HHV-8 infection, even in areas where its prevalence is low.
